# Lack of Plasma Kallikrein-Kinin System Cascade in Teleosts 

**DOI:** 10.1371/journal.pone.0081057

**Published:** 2013-11-20

**Authors:** Marty Kwok-Shing Wong, Yoshio Takei

**Affiliations:** Laboratory of Physiology, Atmosphere and Ocean Research Institute, The University of Tokyo, Kashiwa, Chiba, Japan; University of Patras, Greece

## Abstract

The kallikrein-kinin system (KKS) consists of two major cascades in mammals: “plasma KKS” consisting of high molecular-weight (HMW) kininogen (KNG), plasma kallikrein (KLKB1), and bradykinin (BK); and “tissue KKS” consisting of low molecular-weight (LMW) KNG, tissue kallikreins (KLKs), and [Lys^0^]-BK. Some components of the KKS have been identified in the fishes, but systematic analyses have not been performed, thus this study aims to define the KKS components in teleosts and pave a way for future physiological and evolutionary studies. Through a combination of genomics, molecular, and biochemical methods, we showed that the entire plasma KKS cascade is absent in teleosts. Instead of two KNGs as found in mammals, a single molecular weight KNG was found in various teleosts, which is homologous to the mammalian LMW KNG. Results of molecular phylogenetic and synteny analyses indicated that the all current teleost genomes lack KLKB1, and its unique protein structure, four apple domains and one trypsin domain, could not be identified in any genome or nucleotide databases. We identified some KLK-like proteins in teleost genomes by synteny and conserved domain analyses, which could be the orthologs of tetrapod KLKs. A radioimmunoassay system was established to measure the teleost BK and we found that [Arg^0^]-BK is the major circulating form instead of BK, which supports that the teleost KKS is similar to the mammalian tissue KKS. Coincidently, coelacanths are the earliest vertebrate that possess both HMW KNG and KLKB1, which implies that the plasma KKS could have evolved in the early lobe-finned fish and descended to the tetrapod lineage. The co-evolution of HMW KNG and KLKB1 in lobe-finned fish and early tetrapods may mark the emergence of the plasma KKS and a contact activation system in blood coagulation, while teleosts may have retained a single KKS cascade.

## Introduction

**Figure 1:**
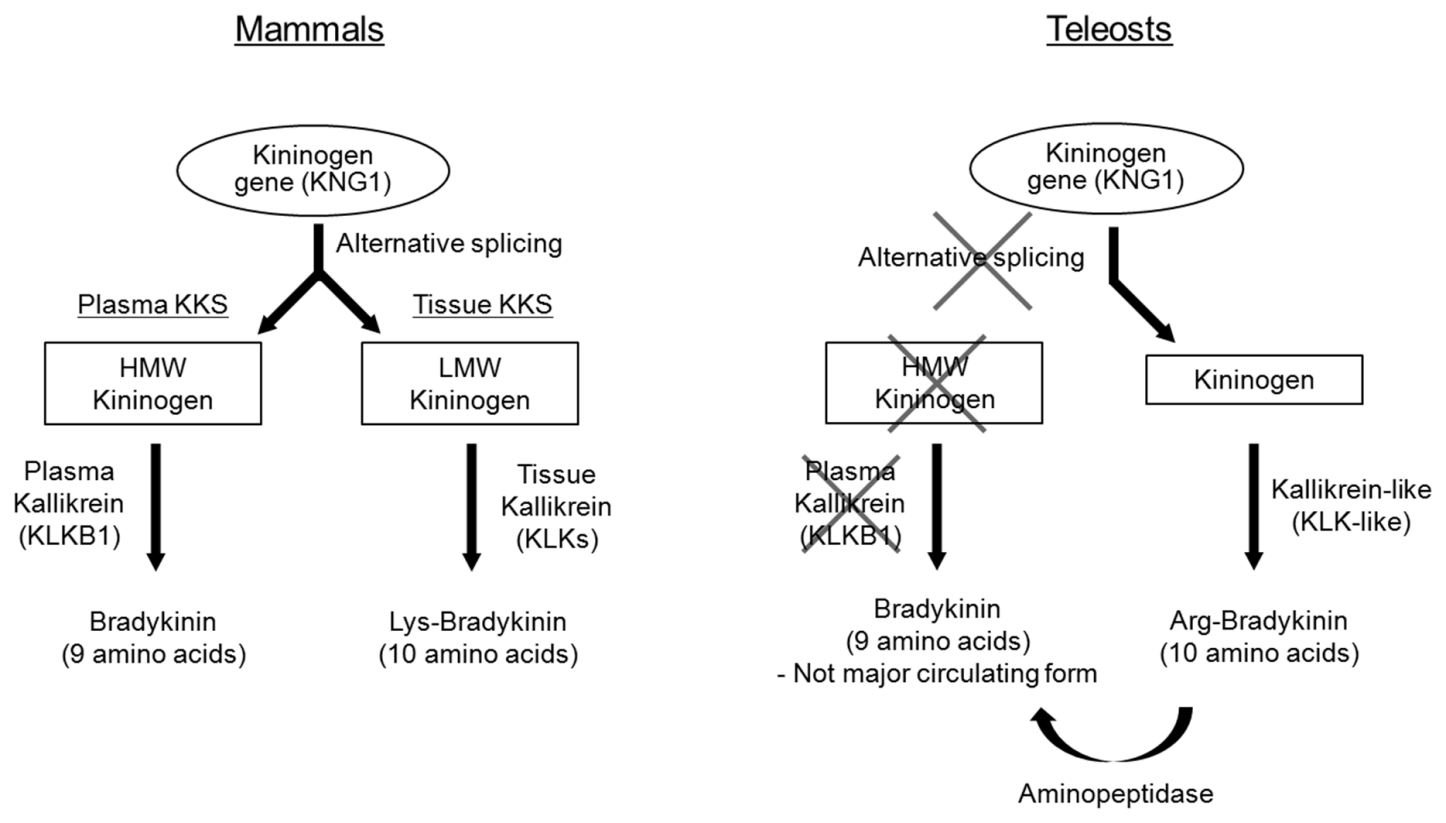
Comparison between the KKS cascade in mammals and teleosts. In mammals, plasma KKS is composed of HMW KNG, KLKB1, and BK; while tissue KKS is composed of LMW KNG, KLKs, and [Lys^0^]-BK. In teleosts, the cascade that is equivalent to the plasma KKS in mammals is entirely absent. KLKB1 was absent in teleost genomes and the nonapeptide BK, which is specifically cleaved by KLKB1, is not the major kinin in the circulation. [Arg^0^]-BK was demonstrated as the major circulating kinin in teleosts and it could be produced by trypsin-like enzymes on the KNG which is equivalent to the mammalian LMW KNG. Since the turnover of kinins is rapid *in* *vivo*, trace amount of BK in the teleost circulation could be the intermediate product of cleavage by aminopeptidase.

The kallikrein-kinin system (KKS) is a conserved set of proteins in vertebrates, which is involved in cardiovascular regulation, inflammation, immune function, pain perception, kidney function, and drinking [1,2]. The functions of kinins are often antagonistic to those of the renin-angiotensin system (RAS) and the two systems often crosstalk at cascade, receptor, and signaling levels [3-5]. Two major cascades, a plasma KKS and a tissue KKS, are the major pathways for the formation of kinins in mammals [6]. In the plasma KKS, high molecular-weight (HMW) kininogen (KNG) is cleaved by plasma kallikrein (KLKB1), to form a nonapeptide known as bradykinin (BK). In the tissue KKS, low molecular-weight (LMW) KNG is cleaved by tissue kallikreins (KLKs) to form a decapeptide called [Lys^0^]-BK or kallidin (see Figure 1 for summary). The HMW and LMW KNGs are products of alternative splicing from the same *kng1*, with the same N-terminal heavy chain but different C-terminal light chains. Tissue kallikreins are serine proteases that were known as glandular kallikreins, but the recent nomenclature system has unified the names and symbols (KLK) of this protease family [7]. BK and [Lys^0^]-BK are short-lived peptides and they act on inducible B1 and constitutive B2 receptors, which are G-protein coupled receptors that modulate concentrations of intracellular calcium, nitric oxide (NO), arachidonic acid, prostaglandins, leukotrienes, and endothelium-derived hyperpolarizing factor [8]. 

Components of the KKS have been identified in various fish lineages including cartilaginous fishes, ray-finned fishes, and lungfish [9-14]. However, a comparative study of the KKS components among fishes and other vertebrates has not been conducted. Instead, our knowledge of the fish systems has been largely extrapolated from mammalian studies. Moreover, since the discovery of KKS in fishes, the endogenous BK types and levels have not been determined due to the lack of a reliable immunoassay. Recently, evidence from genome data suggests that the components of KKS in fishes are different from mammals. These differences included the simple gene structure of *kng1* in fish and lamprey [15,16], and the absence of an orthologous KLK group in the fish lineage [17]. Doolittle [18] suggested that the contact activation system for Hageman factors in blood coagulation as well as plasma kallikrein are absent in fishes, and the enzyme responsible for the formation of BK is not known. Because of the putative difference among the composition of KKS in mammals and fishes, we aim to define systemically the components of KKS in teleosts in the present study. Through biochemical methods, molecular cloning, and genome data-mining, we demonstrated that some of the well-known mammalian KKS components are missing in the teleost lineages. Furthermore, the discovery of the co-evolution of KLKB1 and HMW KNG in a lobe-finned fish has shed light on the evolutionary history and origin of plasma KKS in tetrapods.

## Materials and Methods

### Animal husbandry and sampling

Juvenile, sexually immature Japanese eel (*Anguilla japonica*; 160 - 220 g), tilapia (*Oreochromis niloticus*; 50 - 100g), rainbow trout (*Oncorhynchus mykiss*; 100 - 200 g), and adult medaka of both sex (*Oryzias latipes*; 1.5 - 2.2 g) were kept in a freshwater recirculating aquarium system in the Atmosphere and Ocean Research Institute, the University of Tokyo. The water was maintained at 12 °C for trout, 18 °C for eel, 25 °C for tilapia, and 27 °C for medaka, and all animals were exposed to a 14/10 h light/dark cycle throughout the experiment. All animal experimental procedures were approved by the Animal Experiment Committee of the University of Tokyo. Fish were anesthetized by 0.1% ethyl 3-aminobenzoate methanesulfonate (Sigma, St. Louis, MO) neutralized with sodium bicarbonate. Blood samples were obtained by caudal puncture into syringes containing an inhibitor cocktail (0.05 M 1,10-phenanthroline, 0.225 M potassium EDTA, and 0.1 TIU aprotinin) to prevent clotting and peptide degradation [19]. The v/v ratio of inhibitor cocktails to blood was kept at 0.03 to 1. Blood samples were centrifuged immediately after collection at 10,000 rpm for 5 min at 4 °C and the plasma fraction was obtained and stored at -30 °C until further use. A pooled plasma sample from 10 eel individuals was prepared for validating the immunoassay. 

### Kininogen cloning in eel

Initially, partial sequence of *kng1* in eel was obtained from the draft genome of *Anguilla japonica* (scaffold 7735). The full-length cDNA of eel KNG was obtained using the SMART cDNA Library Construction kit (Clontech Laboratories, Mountain View, CA, USA) according to the manufacturer’s protocol. All sequencing procedures were performed using a BigDye Terminator Cycle sequencing kit and an ABI 3130 DNA sequencer (Life Technologies, Grand Island, NY, USA). N-glycosylation and O-glycosylation sites were predicted using the CBS prediction server [20].

### Distribution of kininogen mRNA in various tissue

 Eel was terminally anesthetized as mentioned previously and various tissues including brain, pituitary, gill, atrium, ventricle, liver, kidney, esophagus, stomach, anterior intestine, posterior intestine, spleen, rete mirabilis, and interrenal were dissected out, snap frozen in liquid nitrogen and stored at -80 °C until use. Total RNA samples were obtained from these tissues, treated with DNase I to remove genomic DNA, and reverse transcribed using SuperScript III First-Strand Synthesis for RT-PCR (Life Technologies, Grand Island, NY, USA). Negative controls for reverse transcription were performed using the same RNA templates in the reactions without reverse transcriptase. The PCR was performed using an ABI 9700 thermal cycler (Life Technologies, Grand Island, NY, USA) with Takara ExTaq DNA polymerase reagents (Takara Bio Inc., Shiga, Japan), and PCR products were electrophoresed on 1.2% agarose gels stained by ethidium bromide. β-actin (*actb*) was used as a positive control to indicate the efficiency and quality of cDNA preparation. The optimal cycle numbers for eel *kng1* and *actb* are 34 and 24 respectively. Primer sequences for eel *kng1* and *actb* are listed in Table S1.

### Development of antisera for [Arg^0^, Trp^5^, Leu^8^]-bradykinin

 [Arg^0^, Trp^5^, Leu^8^]-bradykinin ([Arg^0^]-BK: RRPPGWSPLR) was synthesized by Phoenix Pharmaceuticals Inc. (Burlingame, CA, USA). [Arg^0^]-BK was N-terminally conjugated to thyroglobulin by the glutaraldehyde method. Briefly, 5 mg thyroglobulin was mixed with 1 mg [Arg^0^]-BK in 4 mL PBS (pH 7.0) and glutaraldehyde was added to a final concentration of 0.05 %. The mixture was incubated at 25 °C for 1 h with gentle agitation and reaction was stopped by addition of 0.1 mL ethanolamine. The mixture was dialyzed against 1 L PBS overnight at 4 °C and protein concentration was determined. For each injection, 0.2 mg conjugated protein was used and three rabbits were first immunized with the conjugated protein in Freund’s complete adjuvant followed by two booster immunogen injections in Freund’s incomplete adjuvant. The interval between injections was 4 weeks and the rabbits were sacrificed for final bleeds 3 weeks after the third immunization.

### Western blot of kininogen in teleosts

 Total protein concentrations of the plasma samples from various fish species were determined by a BCA Protein Assay kit (Thermo Scientific, USA). Plasma samples (30 μg protein) were heat denatured under reducing conditions and resolved on a pre-casted 5-20% gradient SDS-PAGE (SPG-520L, Atto Corp., Osaka, Japan) with constant voltage 150 V for 90 min. Resolved proteins were transferred to a 0.45 μm PVDF membrane (GE Healthcare, USA) by a semi-dry western blotting system. The membrane was blocked by 4% skimmed milk in PBS-T (10 mM sodium phosphate, 150 mM NaCl, 0.05% Tween 20, pH 7.4) for 1 h at 25 °C and was subsequently incubated with 1:500 primary antiserum for [Arg^0^]-BK in blocking buffer for 16 h at 4 °C. To displace the bradykinin specific epitope, [Arg^0^]-BK were added to the primary incubation at 10^-5^ M final concentration. After the primary incubation, the membrane was washed three times with PBS-T and incubated with a secondary antibody 1:5000 Anti-Rabbit IgG, horseradish peroxidase-linked (GE Healthcare, USA) in PBS-T. Chemiluminescent signal was produced by Western Lightning ECL reagent (Perkin Elmer, MA, USA) and images were captured on X-ray films (GE Healthcare, USA), which were developed in an in-house darkroom. 

### *In silico* analysis of kininogens, plasma kallikrein, and tissue kallikreins in different vertebrate lineages

 Reference or putative KNG sequences of human, coelacanths, and eel were obtained from Genome databases (NCBI and Ensembl Genome Browser) or by cloning. A list of accession numbers is given in Table S2. Conserved functional domains on these proteins were identified by CDD search on NCBI [21]. Polarity plots of these proteins were constructed according to Zimmerman [22]. Using the coelacanths KNG as a query, a tBLASTn search was performed on the nucleotide and EST databases of Teleostei (taxid:32443) to identify any putative light chain of HMW KNG in fishes. 

Genome search (NCBI and Ensembl Genome Browser) was performed to collect the up-to-date *klkb1* or putative *klkb1* from each representative species under genome project coverage for phylogenetic reconstruction. Homologs of human *klk*s were identified in other vertebrate lineages and possible *klk*-like genes in teleosts were identified according to annotation or predicted synteny relationships. These orthologs of these newly identified *klk*-like genes were used to reconstruct the phylogenetic relationship of *klk*s. Full-length amino acid sequences of various species were aligned using MUSCLE with default setting in Mega version 5, and the best protein model was searched and subsequently used in the phylogenetic analysis. Phylogenetic trees were constructed using the maximum likelihood method (Jones-Taylor-Thornton model with Gamma distribution, categories 5) in Mega version 5 [23]. Bootstrap tests were performed with 100 replicates to check the robustness of the phylogenetic relationships. Synteny analysis was performed among the neighbor orthologous genes of *klkb1* in human, *Xenopus*, coelacanths, medaka, and tilapia to investigate the extent of homology among these orthologs. In *klk* synteny analysis, the orthologs of the neighbor genes of the human *klk*-block were identified in medaka chromosomes and possible *klk*-like genes were identified from uncharacterized or novel proteins. Three dimensional structures of human KLKB1, coelacanths KLKB1, and medaka skin mucus lectin were modeled against the crystal structure of human coagulation factor XI zymogen (2f83A) using a protein structure prediction server [24].

### Development of radioimmunoassay for [Arg^0^, Trp^5^, Leu^8^]-bradykinin

 Synthetic [Tyr^-1^, Arg^0^, Trp^5^, Leu^8^]-bradykinin (YRRPPGWSPLR) was radiolabeled by ^125^I using the lactoperoxidase method and the iodinated peptide was purified by an HPLC system. The ^125^I-BK was diluted in RIA buffer containing 0.1% RIA-grade bovine serum albumin to produce ~10,000 cpm per assay tube. The titers of the antiserum were determined by serial dilution and maximum binding was adjusted at 20 % of the total binding. Incubation of standard [Arg^0^]-BK or unknown samples, radiolabeled tracer, and antiserum (1:10,000) was carried out at 4 °C for 24 h. Separation of free and bound fractions was achieved by precipitation using a secondary antibody, goat anti-rabbit IgG (Sigma, St. Louis, MO, USA), and 16% polyethylene glycol followed by centrifugation at 3,000 g for 1 h at 25 °C and then 30 min at 4 °C. Unbound fractions were removed by aspiration and specific binding in the pellet was measured using a gamma counter (Perkin Elmer, MA, USA). A standard curve that ranged from 3 to 30,000 fmol/mL was constructed. All measurements were performed in duplicate.

### Validation of extraction methods and radioimmunoassay system

The plasma samples were purified by Sep-Pak C_18_ cartridge, with equilibration buffer (100 mM Tris-HCl, 0.5 M NaCl, pH 7.4) for binding and absolute methanol for elution [25]. The partially purified plasma was further resolved by a reverse-phase HPLC system (Tosoh PU-980, Tokyo) attached to a UV-absorbance detector (Tosoh, UV-970, Tokyo) and utilizing an analytical column (ODS 100S column, 4.6 mm I.D. x 25 cm, C-18, 5 μm particle size, Tosoh, Tokyo). A linear gradient from 10% to 60% acetonitrile in 10 mM ammonium acetate, pH 7.0 over 40 min was used for the separation. The column was maintained at 40 °C and flow rate was adjusted to 1 mL/min. HPLC fractions near the elution positions of [Arg^0^]-BK and BK were collected at 1 min intervals and dried by lyophilization. The retention times for [Arg^0^]-BK and BK in the HPLC system were confirmed by the injection of synthetic peptides. The lyophilized fractions were reconstituted in RIA buffer for assay. 

To test the degradation rate of [Arg^0^]-BK in plasma *in vitro*, 3 blood samples with inhibitor cocktail were prepared. Plasma fractions were obtained and divided into 0.2 mL aliquots. Synthetic [Arg^0^]-BK was added to the plasma aliquots to a final concentration of 3,000 fmol/mL and incubated at 25 °C for 0, 10, and 30 min. Incubation was stopped by the addition of equilibration buffer used for Sep-Pak cartridge purification. The immunoreactive (ir)-[Arg^0^]-BK was measured using the newly established RIA. In an additional validation experiment, the pooled plasma samples were spiked by synthetic [Arg^0^]-BK to final concentrations of 300, 1000, 3000 fmol/mL and then extracted as previously stated. The concentration of [Arg^0^]-BK in the HPLC fractions were measured to determine the percentage recovery. Synthetic [Trp^5^, Leu^8^]-BK (BK) and [Arg^0^, Trp^5^, des-Leu^8^, des-Arg^9^]-bradykinin were serially diluted in RIA buffer and measured by RIA to determine the cross-reactivities among these closely related peptides.

## Results

### Western blot of kininogen

**Figure 2:**
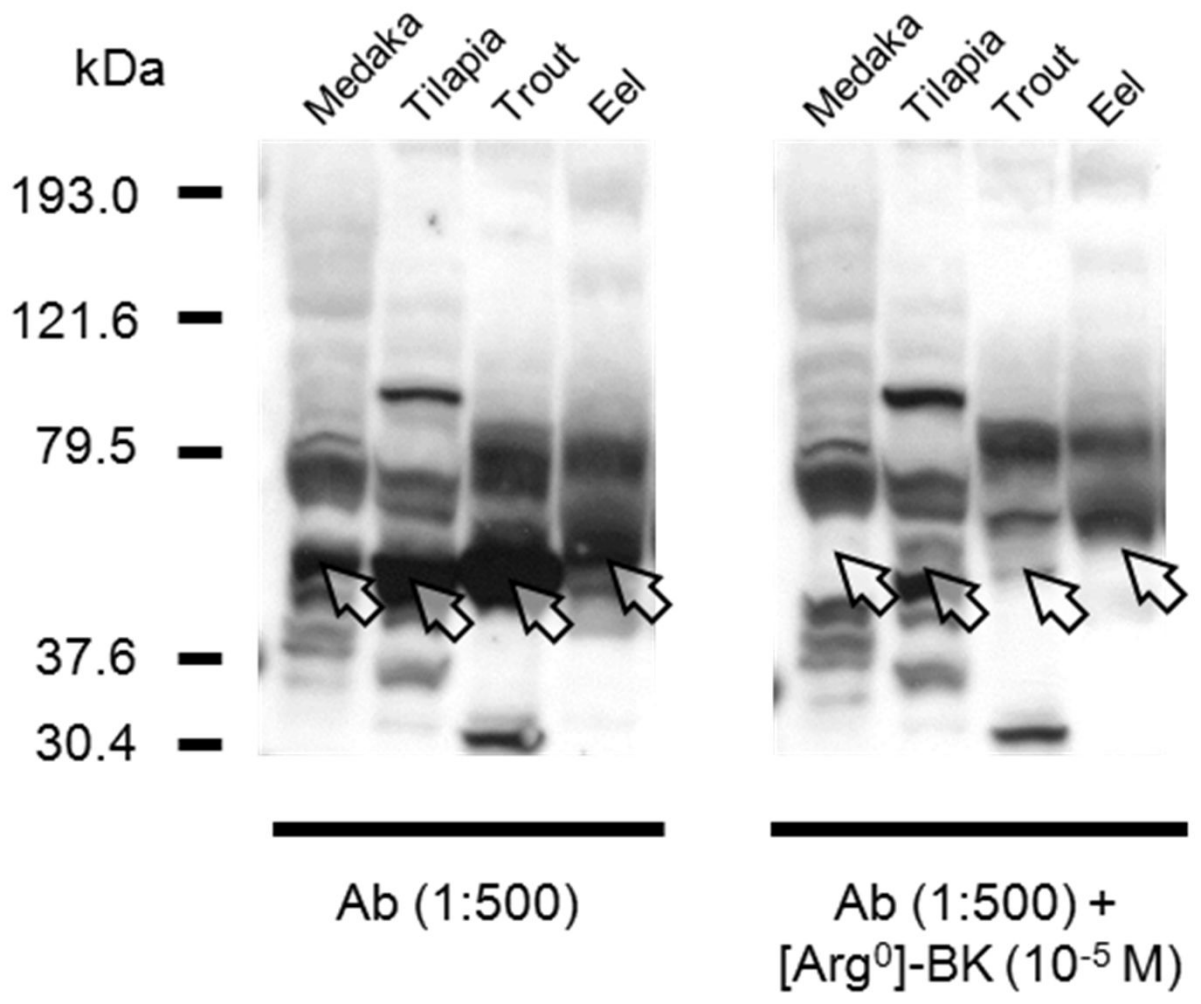
Western blot of KNGs in the plasma of medaka, tilapia, trout, and eel. The antiserum (Ab) recognizes the [Arg^0^]-BK epitope on the linearized KNGs. An immunoreactive band is present at 54-60 kDa in each species, which is completely displaced by the addition of 10^-5^ M [Arg^0^]-BK during the primary incubation. The arrows indicate the corresponding regions where the KNG signals are present (left panel) or displaced (right panel).

 In an attempt to extend the use of the polyclonal BK antiserum on KNG, we tested whether the antiserum can crossreact with the BK epitope on the linearized precursor protein in a western blot system. The ir-KNGs of various fish species were found at 54 - 60 kDa and the signals can be displaced by the addition of excess [Arg^0^]-BK during primary incubation, which demonstrated the specificity (Figure 2). Furthermore, only a single displaceable band was identified in each plasma sample, which suggested that teleosts possess KNG of a single molecular weight instead of HMW and LMW KNGs in mammals. By comparing the observed and predicted molecular weights of KNGs in various teleost species, the percentage glycosylation ranges from 23 - 30%, which is comparable to those of LMW KNGs in mammals (Table 1). 

**Table 1:** Molecular weights (MWs) and glycosylation of kininogens (KNGs) in teleosts.

	Predicted MW (kDa)	Observed MW (kDa)	% glycosylation	References
Medaka	40.6	57.8	29.8	This study
Tilapia	40.3	53.9	25.2	This study
Rainbow trout	41.6	53.9	22.8	This study
Japanese eel	45.0	60.7	25.9	This study
Atlantic cod	38.7	51.0	24.1	[33]
		**mean**	**25.6**	
Human HMW	72.0	120.0	40.0	[34]
Human LMW	47.9	68.0	29.6	[34]

The MWs of teleost KNGs were predicted according to the amino acid sequences. The observed MWs of KNGs were calculated from the western blot (Figure 2). Percentage glycosylation was estimated based on the difference between the predicted and observed MWs. The degree of glycosylation (~25%) of KNGs in teleosts is similar to that of LMW KNGs in mammals.

### Molecular characterization of kininogen gene structure

**Figure 3:**
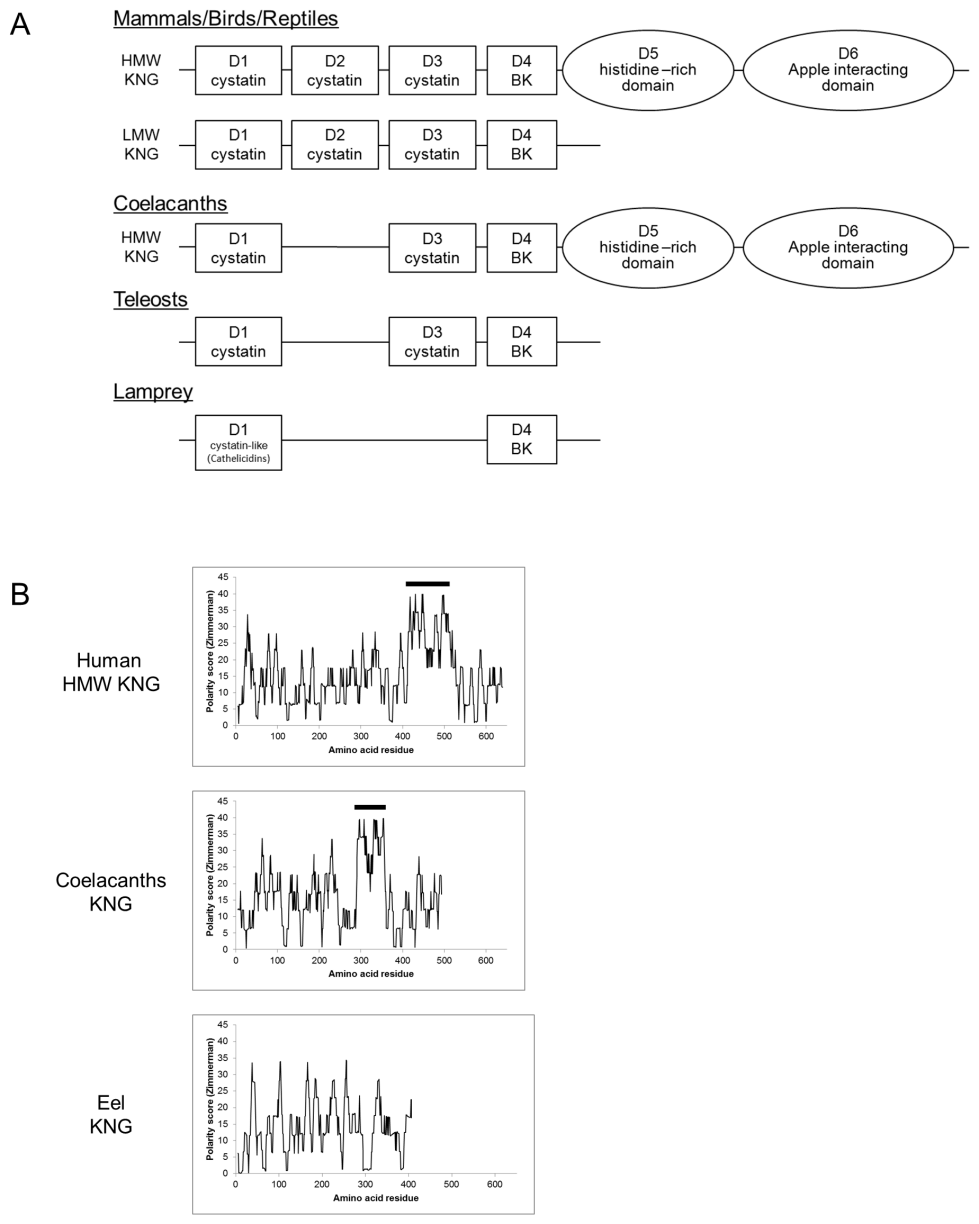
**A.** Schematic diagrams illustrate the various structures of kininogens (KNGs) in vertebrates. In tetrapods, HMW and LMW KNGs are present. HMW KNG possesses three cystatin domains (D1-D3), a BK domain (D4), a histidine-rich domain (D5), and an apple-interacting domain (D6), while LMW KNG lacks D5 and D6. The coelacanths KNG is structurally similar to that of HMW KNG except only two cystatin domains (D1 and D3) are present. The teleost KNGs possess D1, D3, and D4, while the light chain (D5 and D6) is absent. In lamprey, only one cystatin-like (cathelicidin) domain and one BK domain is present. **B.** Polarity blots of the KNGs of human HMW, coelacanths, and eel. The Zimmerman polarity score [22] indicates positively charged domains on the proteins and the unusual positively charged domains are indicated by the horizontal bars. The D5 domain in coelacanths KNG possesses the same charged characteristic and may function in a similar way as in HMW KNGs in tetrapods. No similar positively charged domain is present in teleost KNGs.

 The domain structures of vertebrate *kng1* were compared and summarized in Figure 3A. HMW KNGs in mammals possess three cystatin domains (D1 - D3), a bradykinin domain (D4), a histidine-rich domain (D5), and an apple-interacting domain (D6). LMW KNGs are lacking D5 and D6. An extensive search on the nucleotide and EST database of teleosts also indicated that all examined *kng1* transcripts lack the light chain (D5 and D6) of HMW KNG. The KNG in lamprey consists of one cystatin-like (cathelicidin) domain and a bradykinin domain, which is the simplest KNG structure in vertebrate so far [16]. From conserved domain analysis, the *kng1* in teleosts possess two cystatin domains and a bradykinin domain. Coelacanths KNG possess two cystatin domain, a bradykinin domain, a histidine-rich domain, and a putative apple-interacting domain, which highly resembles the structure of HMW *kng1* in mammals except the absence of D2 (Figure S1). Comparison of polarity plots among human HMW, coelacanths HMW, and eel KNGs indicated that the positively charged D5 is present in coelacanths but not in teleosts (Figure 3B). 

**Figure 4:**
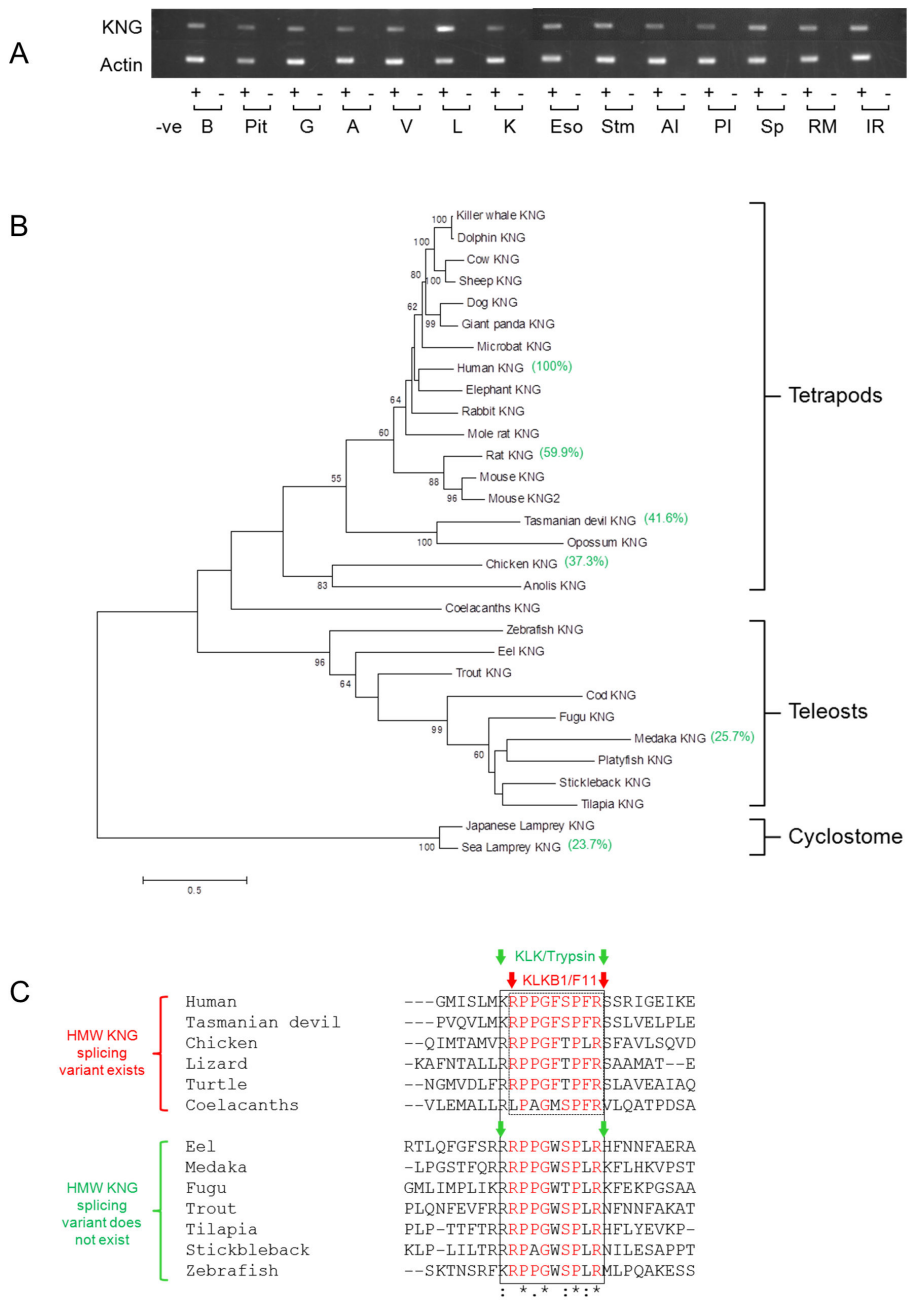
**A.** Distribution of KNG mRNAs in various tissues of Japanese eel. KNG is expressed in most tissues and the highest expression is found in liver. β-actin was amplified to indicate the integrity of cDNA. Sterile water was used as a negative control for the PCR and each RNA sample was reverse transcribed with (+) or without (-) reverse transcriptase. B, brain; Pit, pituitary; G, gill; A, atrium; V, ventricle; L, liver; K, kidney; Eso, esophagus; Stm, stomach; AI, anterior intestine; PI, posterior intestine; Sp, spleen; RM, rete mirabilis; IR, interrenal. **B.** Phylogenetic tree of representative vertebrate KNGs depicted by maximum likelihood method. The deduced proteins of KNGs from the longest transcripts are included in the analysis. The subclade of lamprey KNGs is used as an outgroup to indicate the origin of the tree. Percentage identity matrixes of some representative species are bracketed in green. Numbers on the branches are the bootstrap values (>50%) of 100 replicates. Scale bar represents 50% amino acid substitution. **C.** Amino acid alignment of bradykinin domains in various vertebrate representatives. Conserved amino acids are highlighted in red. KLKB1/F11 is present from coelacanths to tetrapods and it specifically produces a nonapeptide (BK), as indicated by the dashed-line box. KLK or trypsin preferentially cleaves decapeptides ([Lys^0^]-BK or [Arg^0^]-BK) from KNGs. In teleost, KLKB1 is not present and trypsin digestion only results in the formation of [Arg^0^]-BK.

 The eel *kng1* was cloned and sequenced (GenBank accession number: KF111560), and its mRNA distribution in various tissues was shown in Figure 4A. The *kng1* was expressed in all tissues examined and the highest expression was found in liver. Three putative N-glycosylation sites (amino acid residue NXT or NXS provided that X is not a Pro) and 19 putative O-glycosylation sites that cluster at the C-terminal region were identified [20]. The molecular phylogenetic tree of vertebrate KNGs showed that the coelacanths KNG is situated in between tetrapod and teleost KNGs, while teleost KNGs forms a separate cluster (Figure 4B). Using human KNG as a base, percentage identity matrixes of some representative species are shown (in green brackets) to indicate the general protein homology. Cleavage prediction indicates that KLK (trypsin) preferentially produces the decapeptide BK(0-9) in all species investigated while the nonapeptide BK(1-9) of tetrapods is formed by the specific KLKB1-cleavage (Figure 4C). The conserved amino acid residues of BKs in various vertebrates are Pro^2^, Gly^4^, Pro^7^, and Arg^9^. 

### Phylogeny of plasma kallikrein (KLKB1)

**Figure 5:**
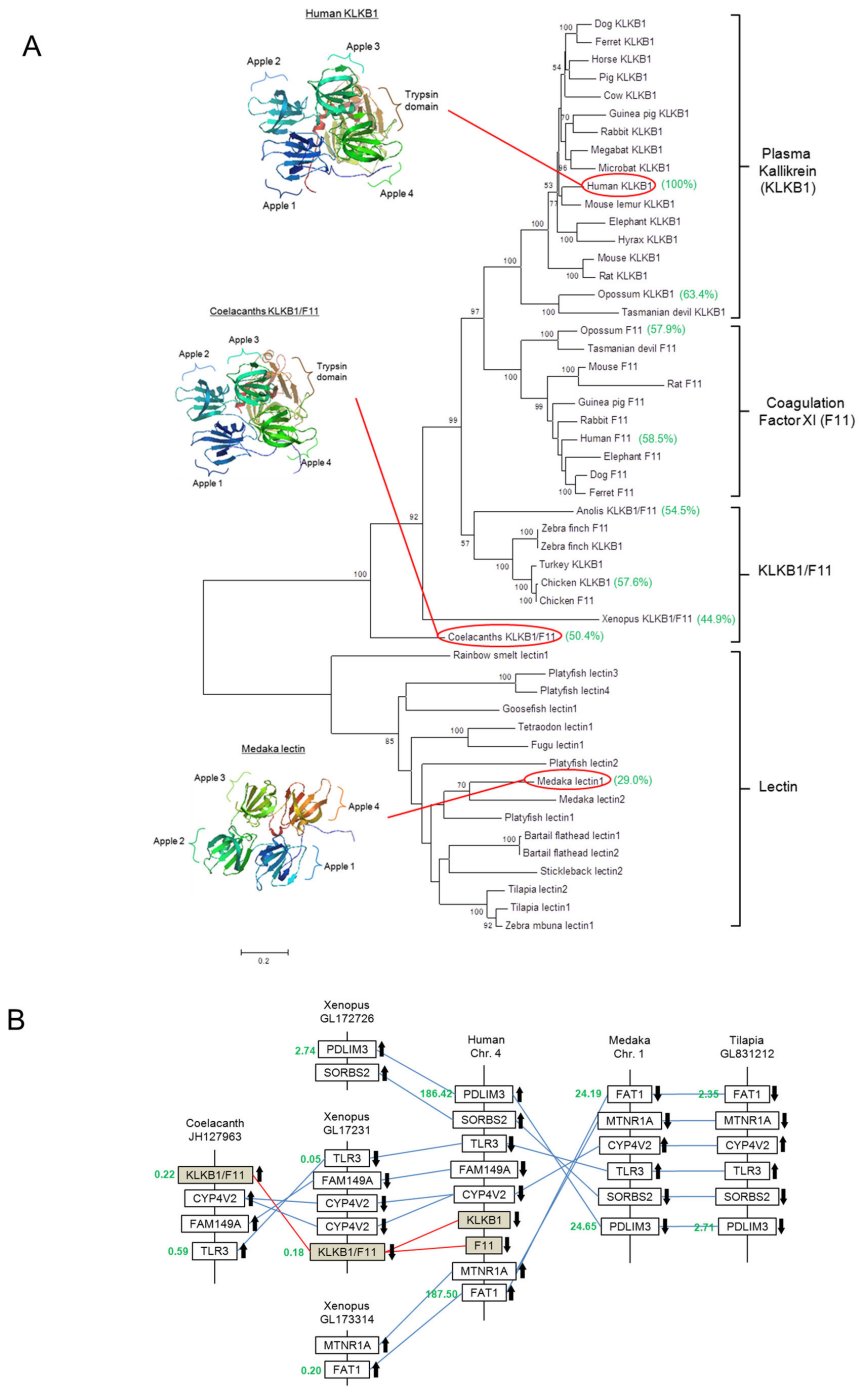
**A.** Phylogenetic tree of representative KLKB1, F11, and teleost lectins depicted by the maximum likelihood method. The coelacanths are the earliest members that possess KLKB1/F11 and the protein is closely related to those of tetrapods while teleost lectins form a separate sub-clade. Three-dimensional modeling showed that the structures of KLKB1/F11 of human and coelacanths share the same basic architecture: four apple domains followed by one trypsin domain. F11 is a duplicated gene of KLKB1 in mammals and they possess the same three-dimensional structure. Teleost lectins possess only four apple domains, but lack a trypsin domain. Percentage identity matrixes of some representative species are bracketed in green. Numbers on the branches are the bootstrap values (>50%) of 100 replicates. Scale bar represents 20% amino acid substitution. **B.** Synteny of the genomic regions containing *klkb1/F11* among coelacanths, *Xenopus*, human, medaka, and tilapia. In the conserved synteny regions, *klkb1/F11* was identified in *Xenopus* and coelacanths but not in medaka and tilapia. Numbers in green indicate the position of the genes (in Mb) on the chromosomes or contigs. The chromosomal directions of the genes are indicated by arrows and the *klkb1/F11* genes are shaded. Connecting lines between chromosomes indicate orthologous genes.

 From the molecular phylogenetic and synteny analyses, *klkb1* is apparently absent in teleost lineage. In the phylogenetic tree (Figure 5A), the earliest vertebrate that possesses *klkb1* is the coelacanths. In mammals, the *klkb1* was tandemly duplicated to form coagulation factor XI (*F11*) and the duplication was specific to the mammalian lineage. Three-dimensional protein modeling of KLKB1 of coelacanths showed the same structural arrangement to that of human: four apple domains followed by a trypsin domain. In teleosts, however, such protein architecture does not exist and the most closely related proteins are some skin mucus lectins, which consist of only four apple domains and are without a trypsin domain. Using human KLKB1 as a base, percentage identity matrixes of some representative species are shown (in green brackets) to indicate the general protein homology. In the synteny analysis (Figure 5B), *klkb1/F11* can be found in mammals, amphibians, and coelacanths, with sets of neighbor genes that show conserved arrangement of gene loci. In teleosts, however, no *klkb1/F11* was found among the corresponding gene loci. Furthermore, the lectin genes in some teleost genomes were annotated as *F11*, which is apparently due to the homology of the four apple domains to *klkb1/F11*. 

### Phylogeny of tissue kallikrein (KLKs)

**Figure 6:**
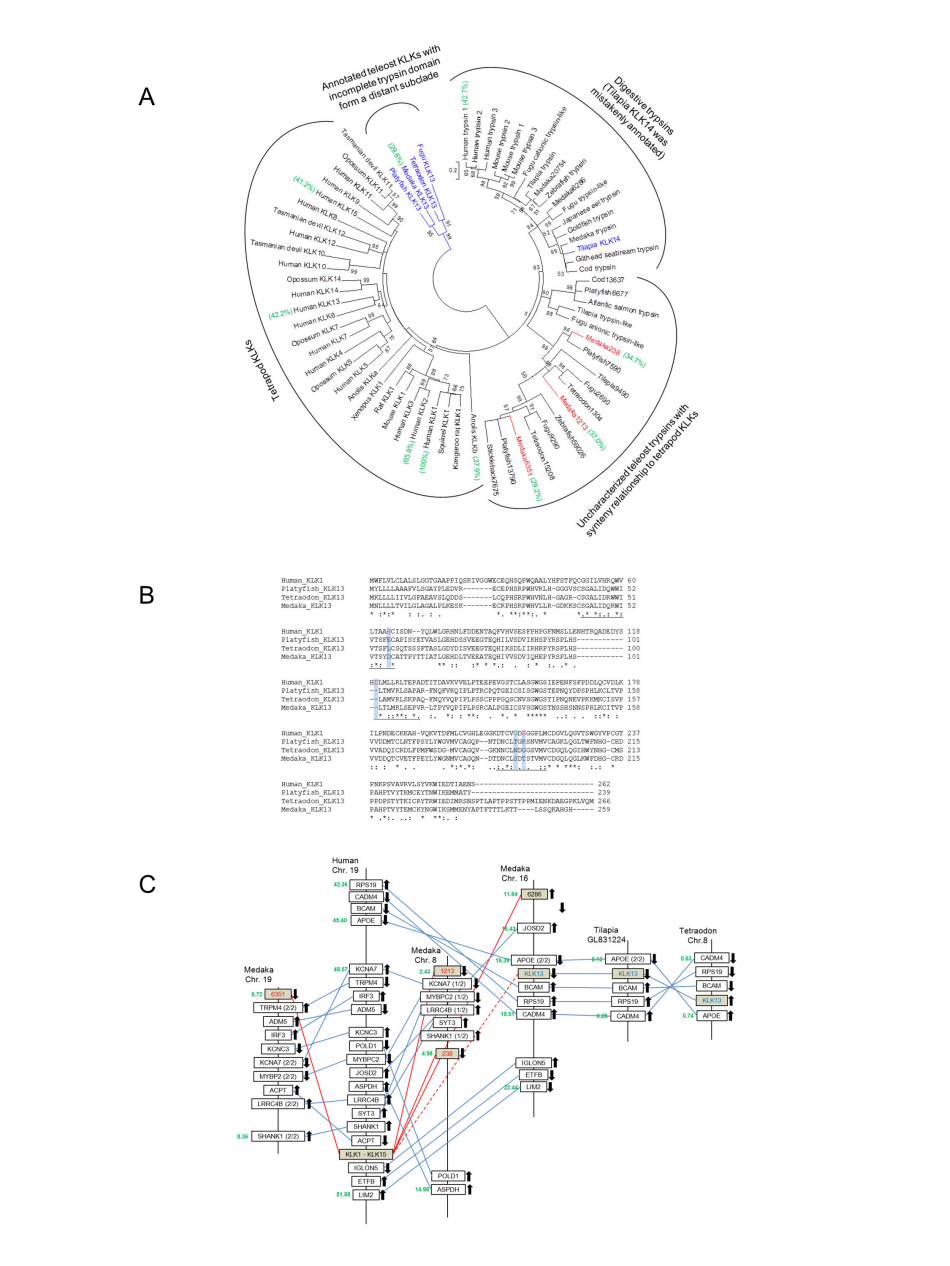
**A.** Molecular phylogenetic tree of KLKs and other putative KLK-like proteins depicted by the maximum likelihood method. In teleosts, KLK13 (highlighted in blue) is annotated but these proteins lack a functional trypsin domain and forms a subclade that is distantly related to the main KLK subclade. Some uncharacterized trypsins with conserved synteny relationship in medaka were identified (highlighted in red and corresponded to the synteny graph in Figure 6C) and these proteins are clustered closely to the KLK subclade of tetrapods. The tree is unrooted. Percentage identity matrixes of some representative species are bracketed in green. Numbers on the branches are the bootstrap values (>50%) of 100 replicates. Scale bar represents 20% amino acid substitution. **B.** Amino acid alignment among human KLK1 and teleost KLK13. The catalytic triads of trypsin domain are underlined and the crucial amino acids are highlighted. The catalytic triads are not conserved in teleost KLK13, which probably indicates that they are not functional trypsins and could be pseudogenes. **C.** Synteny of the genomic regions containing *klks* between human, medaka, tilapia, and *Tetraodon*. In this map, human *klk1-klk15* are shortened to one *klk*-block as they were generated by tandem duplications. The conserved synteny regions on medaka chromosome 8 and 19 are apparently duplicated chromosomes from the 3R. Uncharacterized proteins (named with Ensembl number identifiers) with trypsin domain were identified among the conserved synteny regions in medaka genome and these *klk*-like genes were shaded. Among these uncharacterized trypsins, ENSORLG00000000283, ENSORLG00000001213, and ENSORLG00000006351 (highlighted in red and corresponded to the phylogenetic tree in Figure 6A) form a subclade that is closely related to the tetrapod KLK subclade. Numbers in green indicate the position of the genes (in Mb) on the chromosomes or contigs. The chromosomal directions of the genes are indicated by arrows. Connecting lines between chromosomes indicate orthologous genes. A dashed-line is used for connecting KLK13 (highlighted in blue) as it possesses mutated functional domains.

 Several annotated *klk13* were found in teleost genomes, but the phylogenetic relationship indicated that these sequences are distant from the major *klk* subclade (Figure 6A). Tilapia *klk14* was annotated and we found that this subclade was mistakenly identified and should be classified as a digestive trypsin. Using human KLK1 as a base, percentage identity matrixes of some representative species are shown (in green) to indicate the general protein homology. Alignment of annotated KLK13 of several teleost species with human KLK1 showed that telelost KLK13 lacked the catalytic triads of the trypsin domain (Figure 6B), which suggests that these genes are probably non-functional. Synteny results also showed that the annotated *klk13* in teleosts has a conserved micro-synteny that is not shared by the *klk* block of human (Figure 6C). Among the conserved synteny of *klk*s between human and medaka, we identified several *klk*-like genes from uncharacterized or novel proteins, namely ENSORLG00000000283, ENSORLG00000001213, and ENSORLG00000006351, which possess putative trypsin domains . Alignment of these *klk*-like proteins in teleosts showed that they possess the complete catalytic triads of trypsin, and the phylogenetic relationship showed that they form a separated cluster but could have the same origin as the *klk*s in tetrapods (Figure 6A). 

### Development and validation of bradykinin (BK) radioimmunoassay (RIA)

**Figure 7:**
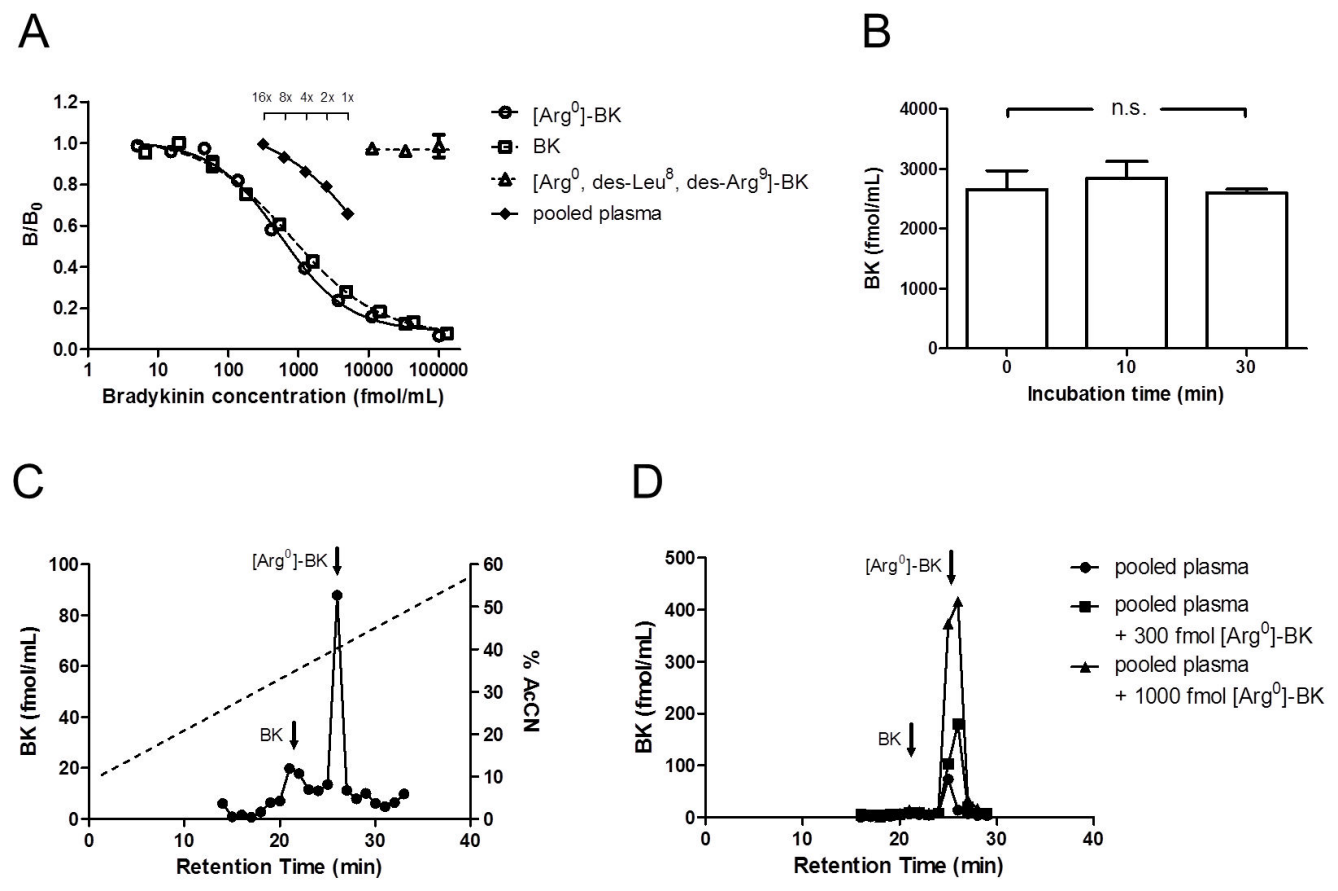
**A.** A representative standard curve for the radioimmunoassay of [Arg^0^]-BK. Synthetic BK exhibits 71.3% crossreactivity while [Arg^0^, Trp^5^, des-Leu^8^, des-Arg^9^]-BK has negligible crossreactivity. The curve produced by serially diluted measurements of a pooled plasma sample is parallel to the [Arg^0^]-BK standard curve. **B.** Integrity of [Arg^0^]-BK in plasma samples. Plasma samples were spiked with 3,000 fmol/mL synthetic [Arg^0^]-BK and incubated for 0, 10, and 30 min before extraction by Sep-Pak cartridge (n = 3). The [Arg^0^]-BK levels are not statistically different among various time points (Student t-test, p > 0.05). Integrity of [Arg^0^]-BK in the samples was protected by inhibitor cocktail, and artefactual BKs are not produced during sample preparation. **C.** A representative ir-BK profile of eel plasma resolved by a reverse-phase HPLC system. The dash-line indicates the HPLC gradient for the separation. The elution positions of [Arg^0^]-BK and BK are identical to those of the synthetic peptide standards. **D.** HPLC-resolved ir-BK of a pool plasma sample spiked with various concentrations of [Arg^0^]-BK. The exogenous [Arg^0^]-BK remained intact in the samples and *in* *vitro* conversion of [Arg^0^]-BK to BK was not observed *in* *vitro*.

 The BK RIA has a EC_50_ value of 550 fmol/mL for [Arg^0^]-BK, and the lowest and highest detection limits are 20 and 20,000 fmol/mL respectively. BK exhibits 71.3% crossreactivity while [Arg^0^, Trp^5^, des-Leu^8^, des-Arg^9^]-BK has negligible crossreactivity (Figure 7A). Serially diluted pooled plasma samples formed a curve that is parallel to that of standard [Arg^0^]-BK. To demonstrate the stability of the assay, we incubated the synthetic [Arg^0^]-BK with plasma samples over a 30 min duration and the treatment did not alter the peptide contents (Figure 7B). A representative chromatograph of ir-BK is shown in Figure 7C, and BK and [Arg^0^]-BK elute at 21 and 27 min respectively. The major circulating form of eel bradykinin is [Arg^0^]-BK but not BK. The synthetic [Arg^0^]-BK spiked in the plasma incubation remained as a single ir-peak after the Sep-Pak/HPLC purification process and the percentage recovery was 51.7% (Figure 7D). The intraassay and interassay coefficients of variation were 4.9% and 13.9% respectively. 

## Discussion

### Kininogens

In humans, HMW and LMW KNGs are products of alternative splicing from the same gene and HMW KNGs possess two extra domains, D5 and D6, which formed the light chain that are absent in LMW KNGs [6]. D5 is a histidine-rich domain which is positively charged (Figure 3B) for interaction with the negatively-charged surface of the endothelium during contact activation in blood coagulation [26]. D6 interacts with the apple domains on KLKB1 and this interaction increases the binding affinity between HMW KNG and KLKB1 [26]. The apple domain is a conserved protein folding with three disulphide bridges, and is often found on diverse proteins for protein-protein or protein-carbohydrate interactions. Both HMW (120 kDa) and LMW (68 kDa) KNGs in human are present in plasma at concentrations of 100 and 90 μg/mL respectively [27]. The antibodies for HMW and LMW KNGs in mammals are usually specific to the C-terminal regions of the proteins, where the two proteins are intrinsically different in structure. In this study, we utilized the same polyclonal BK antiserum used in the RIA system and we found, via western blot, a single KNG that is similar to the mammalian LMW KNGs in various plasma samples in teleosts (Figure 2). Should both HMW and LMW KNGs be present in fish plasma, the antiserum would crossreact with both precursors as the BK epitopes are identical. Although the C-terminal region of [Arg^0^]-BK was used as the immunogen during antiserum production, the polyclonal antiserum is able to recognize the BK epitope of the linearized precursor protein when used at a higher titer (1:500) than that employed for the RIA experiments (1:10,000). The western blot detected a single immunoreactive band at 54 - 60 kDa in each species, a result that demonstrates the lack of HMW KNG in teleosts at the protein level. The percentage glycosylation of KNGs in teleosts was similar to that of LMW KNGs in mammal (Table 1). The expression pattern of eel KNG in various tissues showed combined characteristics of the HMW and LMW KNGs in mammals: a higher expression of HMW KNG in liver that is the major organ for its production and secretion into the circulation; a ubiquitous distribution of LMW KNG in various tissues where the protein is locally cleaved [28]. The expression pattern supports the notion that a single precursor protein in teleost is corresponding to the combined roles of HMW and LMW KNGs in mammals. 

At genomic level, KNG genes in fish lack the D5 and D6 regions [15] and transcripts for the D5 and D6 regions in the nucleotide and EST databases of teleosts were not found, which supports the fact that alternative splicing of *kng1* is non-existent or lost in teleosts. Currently, the evidence favors that the HMW alternative transcript is a shared-derived character introduced in early tetrapods and lobe-finned fish. However, it is also possible that teleosts lost D5 and D6 domain in their own lineage, and the gene structure of cartilaginous fish *kng1* will be an important key to gain insight into the evolution of KNG. 

### Plasma kallikrein

Our results suggest an intimate evolutionary relationship between KLKB1 and HMW KNG in vertebrates as we demonstrated that both these genes are absent in teleosts but first appeared together in coelacanths. As KLKB1 has a specific affinity towards the D6 region of the HMW KNG, coevolution of these two genes is highly probable. Whether KLKB1 existed in the ancestor of fishes and tetrapods cannot be determined at present since no orthologs or pseudogenes of *klkb1* were found in cartilaginous fish or cyclostome databases. The molecular phylogenetic tree of KLKB1 showed that F11 is a duplicated form of KLKB1 and the branch distance suggested that the duplication event occurred in early mammals (Figure 5A). The avian KLKB1/F11 duplication is a separate event and this occurred more recently than that of mammals. The loci of *klkb1* and *F11* in mammals are immediately next to each other, which supports that the origin of these two genes was a result of tandem duplication (Figure 5B). Although it is apparent that *F11* is a mammalian-specific duplicated gene, the sequence similarity of KLKB1 and F11 has caused confusion in the nomenclature of the orthologs in non-mammalian vertebrates. Cross usage of these terms was found in the genome annotations of other vertebrate groups that do not possess F11, and we consider that a united term should be used for non-mammalian lineages in the future. 

KLKB1, which is the specific enzyme for HMW KNG, is not found in any teleost species. KLKB1 is a specific enzyme with unique architecture: four apple domains followed by one trypsin domain. Interaction of D6 to the four apple domains contributes to the high affinity binding and the preference of BK formation instead of [Lys^0^]-BK [26,27]. We showed that *klkb1* is absent in teleost genomes by synteny analysis but whether the absence was due to a deletion or the gene having never evolved remains unknown because no pseudogene of *klkb1* was found among the conserved synteny regions. In teleosts, the skin mucus lectins possess four apple domains but without a catalytic trypsin domain, thus these proteins cannot cleave BK from KNG. Furthermore, the skin mucus lectins were mistakenly considered as plasma kallikrein in the past and the interaction of apple domains with glycans could be involved in immune functions [29,30]. In fact, HMW KNG can be cleaved alternatively to form anti-microbial peptides and the contact phase system in the defense against bacterial infection is connected to innate immunity [6]. Even without a catalytic domain, the lectin with four apple domains could be a candidate protein that binds to the glycosylated KNG, though this relationship remains to be determined 

### Tissue kallikreins

The multiple *klks* that appear as a cluster on the same chromosome are the result of several tandem duplications of a prototypic trypsin in tetrapods. In humans, 15 homologs of *klk*s with trypsin domains were consecutively situated on Chromosome 19, and the evolution of KLKs from amphibians to mammals has been described in detail previously [17]. Among various human KLKs, the percentage identity are relatively low (41.2% - 65.9%), indicating that the duplicated KLK members diversified rapidly in the mammalian lineage. However, the orthologs in the fish lineage have not been studied systemically. Teleost *klk13* was not closely related to the main KLK group (Figure 6A) and have been previously suggested to be unrelated to tetrapod KLKs [17]. The exceptionally long branch lengths of the teleost KLK13 clade away from the main group and the mutated catalytic domain support the pseudogene hypothesis as the loss of function would decrease the selection pressure and thus random mutation would occur more readily (Figure 6A). 

Knowing the mistaken annotation of *klk*s in teleosts, we searched for orthologous *klk* by a synteny approach. Assuming the orthologous *klk* could have a conserved synteny as with tetrapod *klk*s, the genes could be present in teleosts but not yet characterized. Therefore, we searched in the vicinity of conserved gene loci between medaka and human *klk*s, and identified several uncharacterized or novel proteins that could be the orthologs of tetrapod *klk*s. However, it is also possible that the phylogenetically orthologous *klk* does not exist and alternative trypsin genes could have been recruited as the “true kallikrein” in teleosts. The phylogenetic analyses alone cannot determine the “true kallikrein” in the teleosts but the newly identified *klk*-like trypsins could be good candidates to begin with. Further biochemical and physiological experiments are required to establish the correlation between the expression patterns of these *klk*-like trypsins and the status of KKS to determine the native *klk*s in teleosts. 

### Kinins

Judging from the difference in crossreactivities between N-terminal and C-terminal truncated variants in the developed RIA, the polyclonal antiserum has a high affinity and specificity towards the C-terminus of [Arg^0^]-BK (Figure 7A). The circulating levels of kinins in eel were comparable to those of human [31], but instead of BK as the major circulating form, [Arg^0^]-BK is the major circulating kinin in eel. As BKs are notorious for their rapid degradation [31], we tested the sampling and extraction methods by some spiking experiments and found that our protocols are sufficient to inhibit degradation or artefactual production of BKs *in vitro*. From cleavage prediction, trypsin preferentially cleaves a decapeptide instead of nonapeptide from HMW or LMW KNGs, however, the dibasic [Arg^0^-Arg^1^]- or [Lys^0^-Arg^1^]-residues are not cleaved due to the presence of [Pro^2^] [32], which is conserved among different species. On the other hand, the formation of BK, a nonapeptide, is due to specific cleavage by KLKB1, which possesses exceptionally high affinity to HMW KNG [27]. Given the above, the fact that [Arg^0^]-BK is the major circulating kinin in teleosts is unsurprising as we demonstrated that both HMW KNG and KLKB1 are absent. In eel, it was shown that [Arg^0^]-BK is more potent than BK in eliciting cardiovascular and antidipsogenic effects [14], indicating that [Arg^0^]-BK has a greater efficacy than BK to stimulate the endogenous BK receptors in teleosts. Rather than having a physiological role, the residual BK in the circulation could be the result of N-terminal degradation of [Arg^0^]-BK by aminopeptidase as the turnover of kinins is rapid *in vivo* [31]. 

## Conclusion

 Although it is literally incorrect to state the absoluteness of the missing KKS components in any vertebrate group, it is unlikely that the lack of plasma KKS components in multiple nucleotide and genome databases of teleosts is due to pure chance. Comparison between the KKS cascades in mammals and teleost is summarized in Figure 1. We demonstrated that the entire cascade equivalent to that of mammalian plasma KKS is missing in teleosts. The precursor protein (HMW KNG), regulatory enzyme (KLKB1), and active peptide (BK) were all absent, suggesting this cascade was lost or had never evolved in the ancestor of teleosts. Novel findings included demonstration of a single KNG protein in teleost plasma, identification of [Arg^0^]-BK as the major circulating kinin by a newly developed immunoassay, and discovery of a *klk*-like group from uncharacterized proteins in teleost genomes. Since there is no distinction between plasma and tissue KKS, [Arg^0^]-BK could be the sole effector of the KKS in teleosts. The information on the endogenous kinin in teleost is the platform for the future studies of BK receptor affinity and signaling. Coincidently, the earliest vertebrates that possess both HMW KNG and KLKB1 are coelacanths, which imply that the elements of the plasma KKS could have co-evolved in the ancient lobe-finned fishes and descended to the tetrapod lineages. Such intimate relationships in coelacanths could be important for the evolutionary study of the emergence of the contact activation system in blood coagulation. On the other hand, teleosts may have retained a single KKS from the common ancestor, and such simplicity is important for the study of original functions of the KKS in an evolutionary perspective. 

## Supporting Information

Figure S1 **Amino acid alignment between the KNGs of human, coelacanths, and eel.** The absence of D2 in the KNGs of coelacanths and eel is indicated by blue fonts. The red font region indicates the bradykinin domain (D4). Histidine-rich domains (D5) in human and coelacanths are underlined.
(TIF)

Table S1 **Primer sequences for PCR used in tissue expression profiling.**
(TIF)

Table S2 **Accession number for the protein sequences used in phylogenetic analyses.**
(TIF)
