# Supplementary figures and images for: Lack of Plasma Kallikrein-Kinin System Cascade in Teleosts
Source: PLoS One. 2013 Nov 20;8(11):e81057. doi: 10.1371/journal.pone.0081057 (PMC3835742; doi:10.1371/journal.pone.0081057)

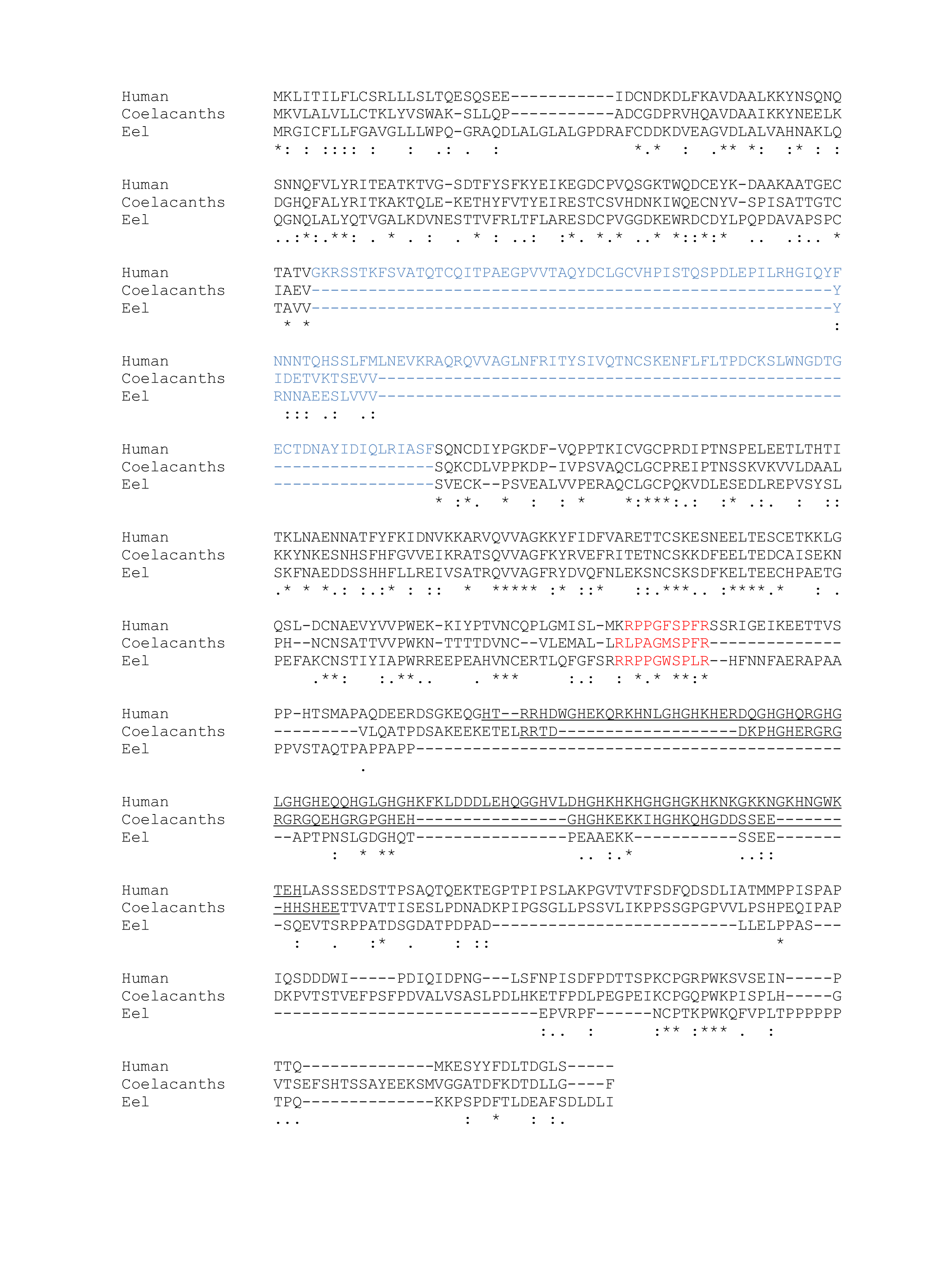

Supplement: Figure S1 — Amino acid alignment between the KNGs of human, coelacanths, and eel. The absence of D2 in the KNGs of coelacanths and eel is indicated by blue fonts. The red font region indicates the bradykinin domain (D4). Histidine-rich domains (D5) in human and coelacanths are underlined. (TIF) [file pone.0081057.s001.tif]
